# Local ice cryotherapy reduced vascular inflammation in large artery from rats with arthritis

**DOI:** 10.1038/s41598-026-41594-0

**Published:** 2026-03-30

**Authors:** Célian Peyronnel, Perle Totoson, Maude Tournier, Francis Bonnefoy, Xavier Guillot, Philippe Saas, Frank Verhoeven, Hélène Martin, Céline Demougeot

**Affiliations:** 1 EFS, INSERM, UMR 1098 RIGHT, Université Marie et Louis Pasteur, 25000 Besançon, France; 2 Service de Rhumatologie, CHU Felix Guyon, Saint-Denis, Ile de la Réunion, France; 3 EFS AuRA, INSERM U1209, CNRS UMR5309, Institute for Advanced Biosciences, Université Grenoble-Alpes, 38000 Grenoble, France; 4 Besancon Cedex, France

**Keywords:** Arthritis, Ice cryotherapy, Endothelial activation, Vascular inflammation, Rat

## Abstract

Rheumatoid arthritis (RA) is associated with increased cardiovascular risk, prompting the exploration of adjunctive therapies to mitigate vascular complications. This study investigated the vascular effects of local ice cryotherapy in the adjuvant-induced arthritis (AIA) rat model, with a focus on vascular inflammation, immune cell infiltration, and arthritis severity. AIA rats received local ice application twice daily to inflamed joints for 14 days. Arthritis severity and joint damage were assessed clinically and radiographically. Aortic mRNA expression of endothelial activation (CXCL-1, CCL-2, CCL-3, ICAM-1, VCAM-1) and dysfunction markers (COX-2, Arginase-2, p22 and p47phox) were assessed by RT-qPCR. Circulating and aortic leukocyte populations (including CD4^+^, CD8^+^, Tc17, Th17 T lymphocytes, neutrophils and monocytes) were determined by flow cytometry. Plasma levels of sclerostin (SOST) and osteoprotegerin (OPG) were measured using Multiplex. Ice cryotherapy significantly reduced arthritis severity and joint damage, decreased aortic COX-2 and p47phox expression, but increased VCAM-1 expression, and markedly reduced aortic infiltration by CD4^+^, CD8^+^ T cells, and Tc17 cells, without affecting circulating leukocyte counts or plasma OPG and SOST. Decrease in arthritis score correlated positively with reduced vascular immune infiltration. These findings suggest potential anti-inflammatory and vasculoprotective benefits from local ice cryotherapy, supporting its use as a well-tolerated RA adjunct therapy.

**Supplementary Information:**

The online version contains supplementary material available at 10.1038/s41598-026-41594-0.

## Introduction

Rheumatoid arthritis (RA) is a chronic systemic inflammatory condition leading to joint damage and physical disability. Although great advances have been made over the last 20 years in the management of RA, life expectancy is still lower than that of the general population due to higher cardiovascular (CV) mortality^1^. The excess CV risk (CVR) in RA patients cannot be entirely explained by traditional CVR factors, suggesting that the systemic inflammation that characterizes RA may cause endothelial disorders leading to a subsequent and accelerated atherosclerosis^2,3^. These endothelial phenotypic alterations are characterized by increased expression of adhesion molecules, enhancement of pro-inflammatory cytokines and chemokines leading to changes in endothelium permeability, endothelial dysfunction and vascular inflammation. Even though the optimal management of CVR in RA is still open to debate, therapeutic interventions in RA, besides controlling disease activity and inflammation, are expected to reduce CVR^1^.

Cryotherapy is a non-pharmacological intervention widely used in RA^4^, that demonstrated benefits on pain relief and disease activity^5,6^. While the interest in whole-body cryotherapy (WBC) is growing, this extreme method is not easy to perform in ambulatory practice and has various contraindications including several CV diseases. Moreover, data indicated that, at least for pain reduction, WBC was not superior to local cryotherapy in RA patients^7^. Thus, local cryotherapy appears as a relevant adjunct therapy in RA, especially if CV diseases are present. Local cryotherapy consists in application on the joints of ice or cold packs (inducing progressive and prolonged cooling), or cold gases (inducing more ample and brutal temperature drops). The possibility of differential effects between these various modalities of local cryotherapy in RA is still an unresolved question. Studies comparing the effect of a 1-day local cryotherapy in patients with knee arthritis reported no difference between ice or cold gas regarding synovial doppler activity, pain^8^ or synovial levels of metabolites involved in energy metabolism^9^. By contrast, differences were observed with respect to the reduction in synovial levels of inflammatory mediators, with a better effect of ice compared to cold gas^10^. As regards knee joint proprioception or analgesia, some differences were observed between ice and cold^11,12^, with no clear conclusions about the superiority of one over the other. Regarding the systemic anti-inflammatory effects of different modalities of cryotherapy, a study conducted in the model of rat adjuvant-induced arthritis (AIA) showed that ice (for 30 min) or cold gas (for 2 min) applied on paws twice a day for 14 days both reduced arthritis severity but only ice decreased plasma levels of IL-6. The systemic anti-inflammatory effect of local cryotherapy raised the hypothesis that this strategy might have positive effects on organs remote from the joint, notably the CV system. Whereas local cold spray therapy was reported to decreased vascular inflammation in AIA rats^13^, no data exist on ice cryotherapy.

The aim of the present study was to investigate if a subchronic treatment with a local cryotherapy using ice applied on paws had positive vascular effects. Experiments were conducted in the model of rat AIA, a reference model for the studies of RA-associated CV disorders^14^. Cytokines expression and leucocyte infiltration were explored in aortic tissue, arthritis severity was determined by clinical and radiographical scores, and peripheral immune activation was assessed through blood leucocytes count. To understand the link between bone and vascular system after cryotherapy, plasma levels of osteoprotegerin (OPG) and sclerostin (SOST) were measured.

## Methods

### Animals and experimental groups

Experiments were conducted on six-week-old male Lewis rats (JANVIER LABS, Le Genest-Saint-Isle, France). Animals were housed under a 12 h light/12 h dark cycle with ad libitum access to food and water. This study was conducted in accordance with national and international regulations on the protection of animals used for scientific purposes, including the European Directive 2010/63/EU. All experimental procedures were approved by the local ethics committee “Comité d’Ethique Bisontin en Expérimentation Animale, CEBEA#58” for animal experimentation (Approval No. 2019-003-PT-5PR, Université Marie et Louis Pasteur, Besançon, France) and were conducted in accordance with the “Animal Research: Reporting In Vivo Experiments” (ARRIVE) guidelines, to ensure animal welfare and minimizing pain and distress. Rats were randomly assigned to two experimental series: series 1 (n = 6–12 rats per group) was used to assess aortic endothelial markers of inflammation, activation, and dysfunction, while series 2 (n = 9–10 rats per group) was used for quantification of leukocyte counts in the aorta and blood. Variations in sample size between series resulted from predefined exclusion criteria related to tissue quality and technical feasibility of the analyses.

### Induction and clinical evaluation of the arthritis model

Arthritis was induced by a single intradermal injection at the base of the tail of 120 µL containing 1 mg of heat-killed *Mycobacterium butyricum* (DIFCO, Detroit, MI, USA) suspended in 0.1 mL of mineral oil (Freund’s incomplete adjuvant; DIFCO, Detroit, MI, USA). Control animals received 120 µL of sterile saline solution. This model typically develops a severe polyarthritis, with clinical signs appearing around day 10 post-immunization, peaking between days 20 and 24, and then progressively declining until stabilizing at a low level by day 60 as previously described^13,15^**.** Rats were monitored daily for body weight and clinical signs of arthritis. Arthritis severity was assessed using a standardized scoring system: inflammation of a single digit was scored as 0.1; mild to moderate arthritis of a major joint (ankle or wrist) was scored as 0.5; and severe arthritis of a major joint was scored as 1. The tarsus and ankle were considered as a single joint. The cumulative score across all four limbs yielded a maximum possible arthritis score of 6 per rat.

### Ice cryotherapy protocol

Local ice cryotherapy was applied twice daily at 8-h intervals (9:00 A.M. and 5:00 P.M.) for 14 consecutive days, following the protocol described by Guillot et al.^16^. Cages (housing two rats separated by ice chips) were lined with 50 mL ice pops (YETI, YETIGEL, Avignon, France) previously frozen at − 26 °C. Rats were placed individually in the ice-lined cages for a 30-min session. Rats were placed in the cages and removed 30 min later at a 5 min-interval in an alternating order. Room and skin temperatures were monitored using an MLT409/A skin temperature probe connected to an ML309 transducer Thermistor Pod (AD INSTRUMENTS). The skin temperature of the tarsus and ankle of each hind paw was measured immediately after each cold application. This protocol resulted in a decrease in hind paw skin temperature from 28.3 ± 0.3 °C to 18.4 ± 0.4 °C at the end of each session.

### Tissue collection

Tissue collection was performed the day following the final cryotherapy session. All rats were anesthetized by intraperitoneal injection of sodium pentobarbital (60 mg/kg, EXAGON, AXIENCE S.A.S., Pantin, France) and euthanized by total exsanguination via the abdominal artery. Thoracic aortas of rats from series 1 were excised, cleaned, snap-frozen in liquid nitrogen, and stored at − 80 °C for subsequent qPCR analysis. In rats from series 2, thoracic aortas and whole blood were collected and immediately processed for flow cytometric analysis. A blood fraction was centrifuged and the plasma stored for later measurement of OPG and SOST levels. Hind paws were harvested and fixed in 4% formalin.

### Radiological examination and radiographic score assignment

Joint damage was assessed by X-ray radiography using a BMA High-Resolution Digital X-Ray system (40 mV, 10 mA; D3A MEDICAL SYSTEMS), following a previously established protocol in Peyronnel et al.^13^. Each hind limb was scored on a scale from 0 to 40 based on five parameters: swelling, cartilage destruction, osteoporosis, bone erosion, and new bone formation, according to the modified rating scale described by Ackerman et al.^17^.

### RT-qPCR analysis

The mRNA expression of endothelial activation (adhesion molecules, cytokines, chemokines) and endothelial dysfunction-related markers (involved in nitric oxide metabolism, oxidative stress, and cyclo-oxygenase pathways)^18^ were measured in thoracic aortas as previously described in Peyronnel et al.^13^. Briefly, total RNA was extracted using the RNeasy Fibrous Tissue Mini Kit (QIAGEN, Hilden, Germany), and 0.5 µg was reverse transcribed using the iScript cDNA Synthesis Kit (BIO-RAD LABORATORIES, Hercules, USA). Quantitative PCR was performed with the iQ SYBR Green Supermix (BIO-RAD) on a iCYCLER THERMAL CYCLER 582BR (BIO-RAD). Primer sequences for target genes related to endothelial activation (ICAM-1, VCAM-1, CXCL-1, CCL-2, CCL-3)^19^, endothelial dysfunction^18^ (Arginase-2, COX-2, p22 phox and p47 phox), and reference genes (β-actin, GAPDH) are listed in Table 1.

**Table 1:** Primer sequences used in qRT-PCR.

Target	Forward primer (5′ → 3′)	Reverse primer (5′ → 3′)
Arg-2	CTC-TGG-ATC-TTG-TTG-AAG	ACT-TGA-AGC-AAT-CAC-ATC
ICAM-1	TGC-CTG-CAC-TTT-GCC-CTG-GT	ACA-GGC-CCG-GGG-ATC-ACA-AC
VCAM-1	TTG-TTC-AAG-AGA-AAC-CAT-TTA-GTG-T	TCA-TCC-TCA-ACA-CCC-ACA-GG
CXCL-1	CCA-GCC-ACA-CTC-CAA-CAG-AGC-A	GGC-GCC-CCT-GTG-GCT-TGG-
CCL-2 (MCP-1)	GTG-TGA-TTT-GGA-ATG-TGA-TG	AAG-TGT-TGA-ACC-AGG-ATT
CCL-3 (MIP-1α)	AGA-ACA-TTC-CTG-CCA-CCT	AAG-TGA-AGA-GTC-CCT-GGA-T
COX-2	TTT-GCC-TCT-TTC-AAT-GTG	TTA-ATG-TCA-TCT-AGT-CTG-GA
P22 phox	ACC-TGA-CCG-CTG-TGG-TGA-A	GTG-GAG-GAC-AGC-CCG-GA
P47 phox	TCC-TAT-CCC-TAC-CCT-TGT	GAG-TCT-GAG-TCC-ATT-CCA
β-Actin	TAT-CGG-CAA-TGA-GCG-GTT-GC	TGC-CTG-GGT-ACA-TGG-TGG-TG
GAPDH	GGG-CAT-CCT-GGG-CTA-CAC-TG	GAG-GTC-CAC-CAC-CCT-GTT-GC

All the samples were deposited in duplicates. Each plate included two negative controls: RNAse-free water and no-RT controls. Thermocycling conditions consisted of an initial polymerase activation step at 95 °C for 3 min, followed by 40 cycles of 95 °C for 15 s and 60 °C for 60 s. Fold changes between groups were calculated with MYIQ SYSTEM Software v1.0.410 (BIO-RAD, https://www.bio-rad.com/fr-fr/product/firmware-software-updates?ID=fb6f1fd4-fd6d-4715-87c7-ec8d429fcf41) using normalized and averaged fluorescence ratios of target genes in samples from the different rat groups.

### Flow cytometry

The leukocyte populations present in blood and infiltrated in aortas were analyzed by flow cytometry as previously described in details in Peyronnel et al.^13^. Briefly, cells extracted from digested aorta were counted and then prepared for flow cytometry with the following antibody mix: CD45 BV510, CD3 APC, CD11b/c BV650, CD4 PE-Cy7, CD8 BV7711, PE granulocytes (RP-1 Antigen) (BD BIOSCIENCES, Le Pont-de-Claix, France).

Blood analysis was performed using Trucount tubes (BD BIOSCIENCES) with the same antibody panel. To assess IL-17A-producing lymphocyte subpopulations in blood and aorta, intracellular cytokine staining was performed following stimulation with phorbol myristate acetate (PMA, 1 µg/mL) and ionomycin (25 ng/mL) in the presence of GolgiPlug (BD BIOSCIENCES). After 4 h of stimulation, cells were stained with surface antibodies to identify leukocytes, lymphocytes, CD4^+^ and CD8^+^ T cells (CD45 BV510, CD3 APC, CD4 PE-Cy7, CD8 BV711, respectively). Cells were then permeabilized (Cytofix/Cytoperm, BD BIOSCIENCES) for intracellular labeling with FITC-conjugated anti-IL-17A monoclonal antibody (BD BIOSCIENCES). Samples were analyzed on a BD LSR Fortessa flow cytometer. Data are expressed as the number of labeled cells per mg of thoracic aorta or per μL of blood. The flow cytometry gating strategy is detailed in Supplementary Fig. 1.

### Plasma levels of bone-related proteins

Plasma levels of OPG and SOST were measured. These two regulators of bone metabolism have been proposed as biomarkers of CV diseases, notably in patients with RA^20,21^. SOST and OPG levels were measured in plasma using Milliplex magnetic bead panel kits (3plex SPR2285 for SOST and RBN1MAG-31 K for OPG, MERCK, Darmstadt, Germany) and then analyzed with a MAGPIX system (LUMINEX CORPORATION, Austin, USA). The limit of detection was 2.44 pg/mL for SOST and 1.3 pg/mL for OPG.

### Data and statistical analysis

Values were expressed as means ± SEM. Data were analyzed using GRAPHPAD PRISM Software v8.0.1 (https://www.graphpad.com/updates). Data normality was assessed using the Shapiro–Wilk test. When data were normally distributed, comparisons between groups were performed using an unpaired Student t test. In most cases, data were not normally distributed, and therefore the non-parametric Mann–Whitney test was applied. Relationships between variables were assessed using Spearman’s rank correlation coefficient. A *p*-value < 0.05 was considered statistically significant.

## Results

### Ice cryotherapy alleviated clinical arthritis and radiographic damage

**Fig. 1:**
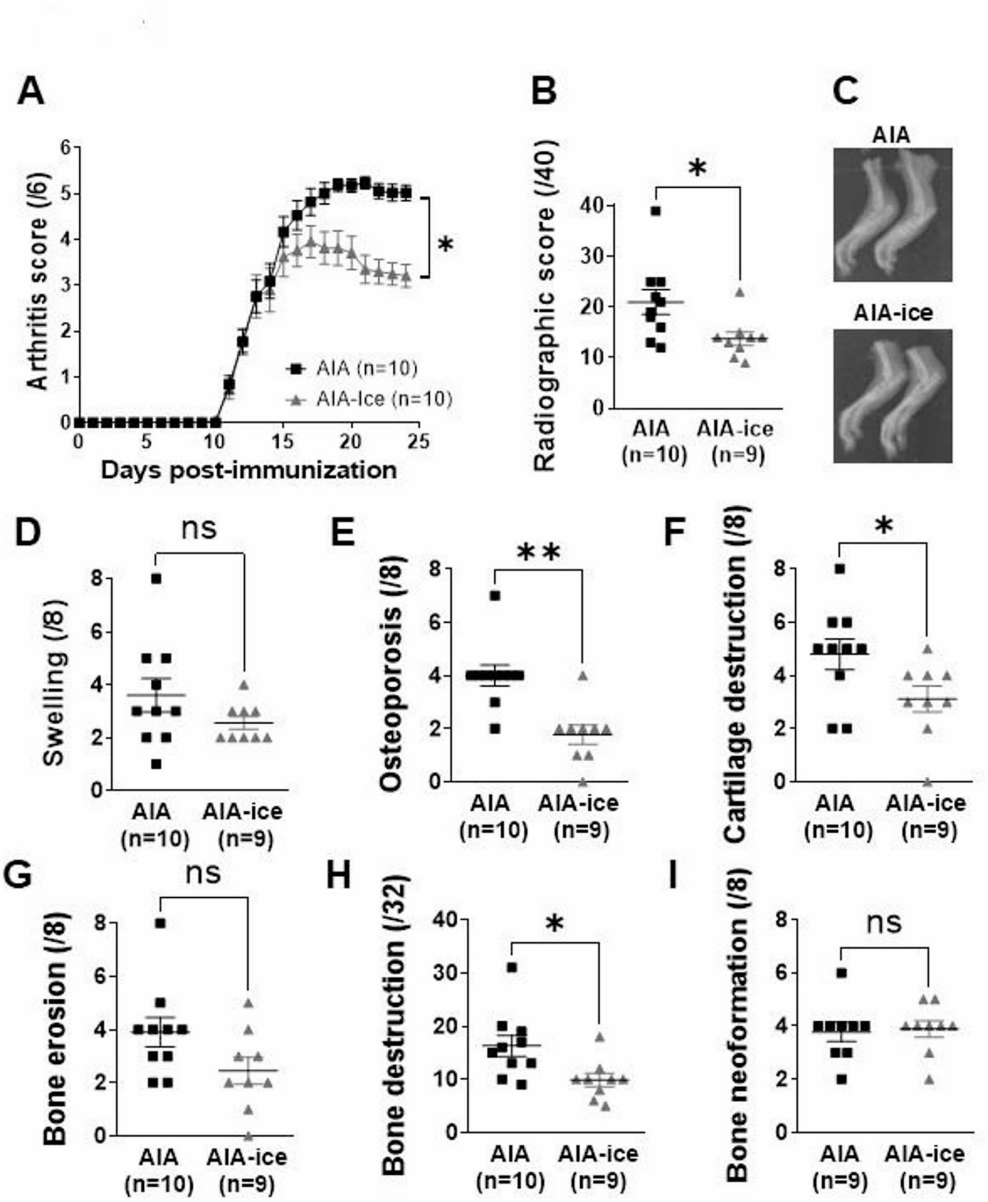
Local ice cryotherapy reduced arthritis severity and joint damage in AIA rats. Experiments were conducted in AIA rats treated or not with daily local ice cryotherapy from day 11 to day 24 post-immunization. (**A**) Time-course of arthritis score. (**B**) Radiographic score and (**C**) representative X-ray radiography of hind paws at day 24. (**D**–**I**) Effect of ice cryotherapy on each item of the radiographic score: swelling, cartilage destruction, osteoporosis, bone erosion and new bone formation. Results are expressed as means ± SEM (n = number of rats/group). *(*p* < 0.05), **(*p* < 0.01).

At the end of the treatment period, local ice cryotherapy reduced the arthritis score by 36% and the radiographic score by 34% compared to untreated AIA rats (Fig. 1A–C). Further analysis of the items of the radiographical score revealed that ice cryotherapy predominantly reduced osteoporosis, cartilage and bone destruction (Fig. 1D–I).

### Ice cryotherapy hampered endothelial dysfunction and reduced immune cells infiltration in aorta

**Fig. 2:**
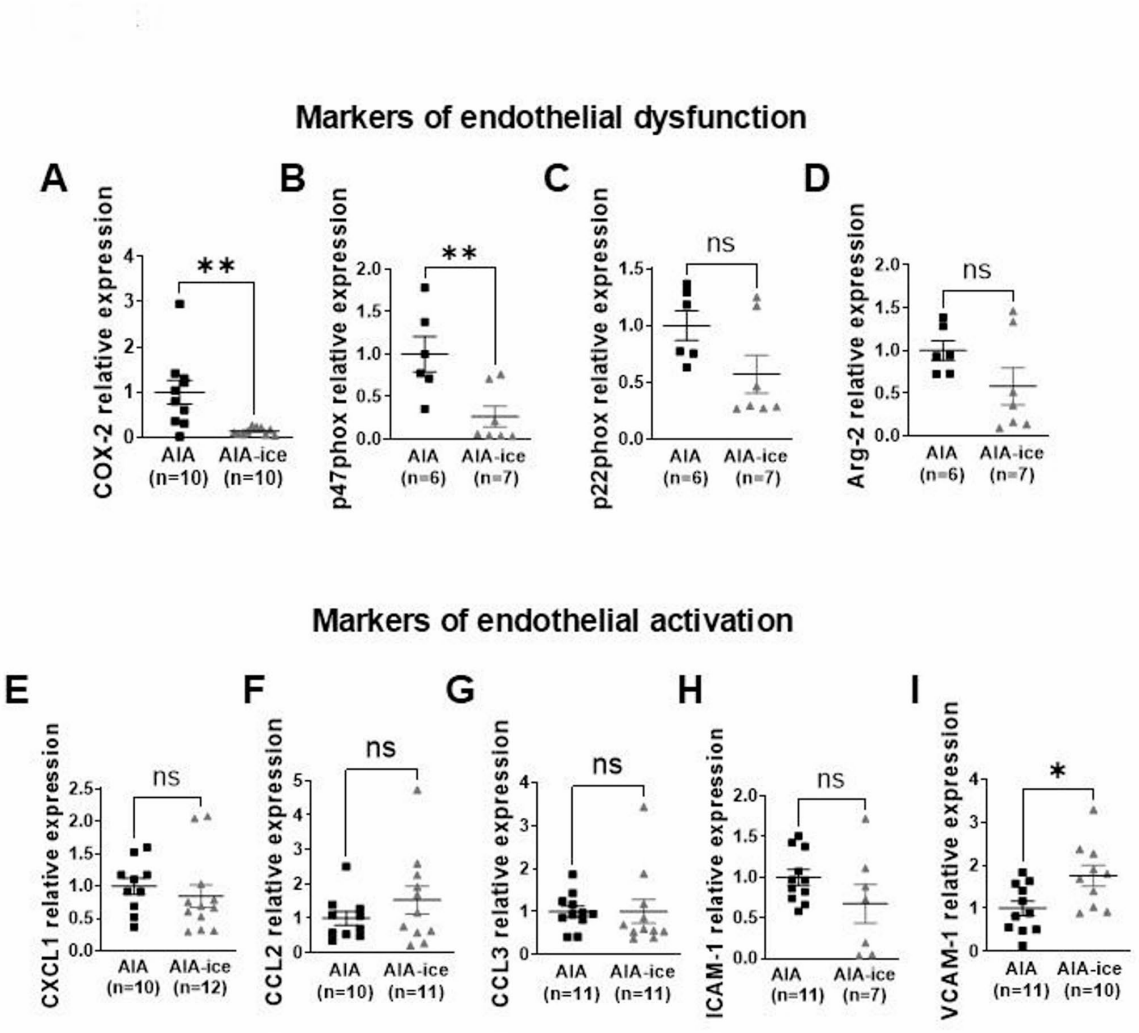
Effect of local ice cryotherapy on endothelial activation/dysfunction markers in aorta from AIA rats. Aortic expression of mRNA of various markers of endothelial activation and dysfunction was measured in AIA rats treated or not with daily local ice cryotherapy from day 11 to day 24 post-immunization by qRT-PCR. (**A**–**D**) mRNA expression of markers of endothelial dysfunction: COX-2, P47phox, p22phox and arginase-2 (Arg-2). (**E**–**I**) mRNA expression of markers of endothelial activation: CXCL-1**,** CCL-2**,** CCL-3**,** ICAM-1, VCAM-1. Results are expressed as means ± SEM (n = number of rats/group). *(*p* < 0.05), **(*p* < 0.01), ns: non-significant.

**Fig. 3:**
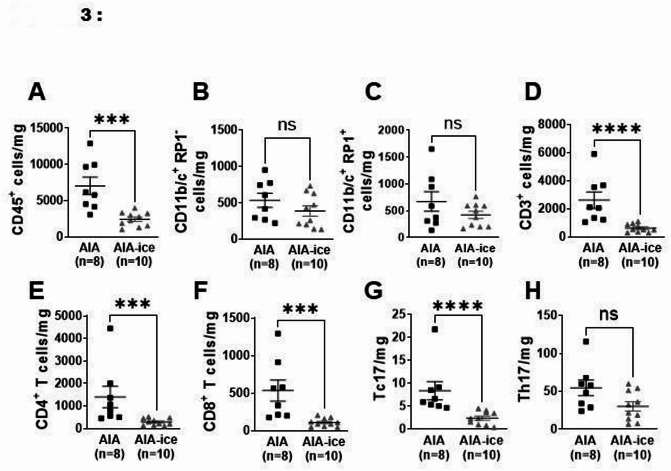
Local ice cryotherapy reduced aortic wall leukocyte infiltration in AIA rats. Effect of local ice cryotherapy in AIA rats treated from day 11 to day 24 post-immunization on aortic count of total leukocytes CD45^+^ (**A**), and different subpopulations: monocytes/macrophages CD11b/c^+^ RP-1^-^ (**B**), neutrophils CD11b/c^+^ RP-1^+^ (**C**), T lymphocytes CD3^+^ (**D**), CD4^+^ (**E**), CD8^+^ (**F**), CD4^+^ IL-17A^+^ (**G**) and CD8^+^ IL-17A^+^ (**H**) T cells. Gating strategies are shown in Supplementary Fig. 1A–J. Data are presented as number of stained cells per mg of aorta. Results are expressed as means ± SEM (n = 8–10 rats/group). *(*p* < 0.05), **(*p* < 0.01), ***(*p* < 0.001), ns: non-significant.

In aorta, as compared to untreated AIA, ice cryotherapy drastically reduced COX-2 and p47phox relative expressions (Fig. 2A,B, whereas the reduced expression of arginase 2 and p22phox did not reach significance (Fig. 2C,D). The mRNA levels of endothelial activation markers were not influenced by the treatment (Fig. 2E–H), except VCAM-1 whom relative expression was increased (Fig. 2I). Consistent with the decrease in vascular inflammation, ice cryotherapy markedly reduced total leukocytes aortic infiltration, with a particular effect on T cells and more specifically on CD4^+^, CD8^+^ T and Tc17 cells (Fig. 3A,D–G). On the other hand, despite a downward trend, ice cryotherapy showed no significant effect on aortic neutrophil (CD11b/c^+^ RP-1^+^) and monocyte/macrophage (CD11b/c^+^ RP-1^−^) populations, nor on Th17 lymphocytes (Fig. 3B,C,H). Of note, arthritis score positively correlated with aortic infiltration by total leukocytes (CD45^+^) (r = 0.5049 ; *p* = 0.0326, n = 18), total T cells (CD3^+^) (r = 0.5887; *p* = 0.0102 n = 18), CD4^+^ T cells (r = 0.5235; *p* = 0.0258 n = 18), Th17 (r = 0.5352 ; *p* = 0.0221 n = 18) and CD8^+^ T cells (r = 0.5442; *p* = 0.0195 n = 18).

### Ice cryotherapy did reduce neither the number of circulating leukocytes nor plasma levels of bone-related proteins

**Fig. 4:**
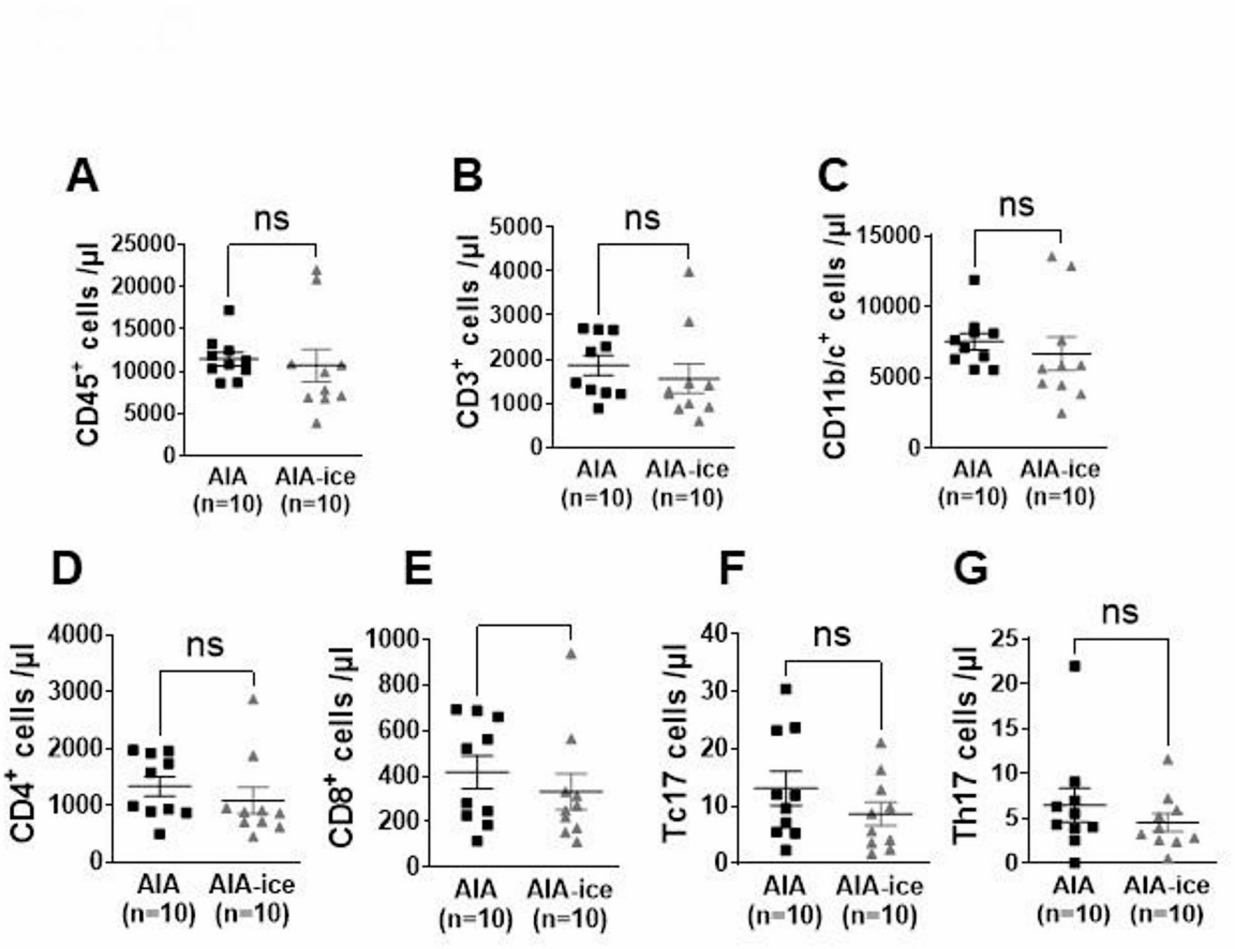
Ice cryotherapy did not change circulating leukocytes count in AIA rats. Effect of local ice cryotherapy in AIA rats treated from day 11 to day 24 post-immunization on blood leucocytes and leukocyte subpopulations in AIA rats. Flow cytometry analysis assessed the absolute number of blood leukocytes (CD45^+^ cells, (**A**) and of the different leukocyte subpopulations: CD3^+^ T lymphocytes (**B**), CD11b/c^+^ monocytes (**C**), CD4^+^ (**D**), CD8^+^ (**E**) T cells, and intercellular IL-17A^+^ CD4^+^ (**F**) or CD8^+^ (**G**) T cells. Gating strategies are shown in Supplementary Fig. 1K–R. Data are expressed as number of stained cells per µL of blood. Results are expressed as means ± SEM (n = 8–10 rats/group). ns: non-significant.

**Fig. 5:**
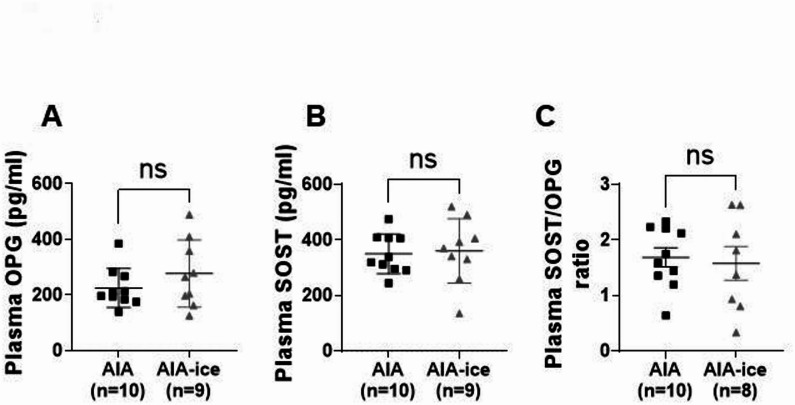
Ice cryotherapy did not change circulating levels of OPG and SOST in AIA rats. Experiments were conducted in AIA rats treated or not with daily local ice cryotherapy from day 11 to day 24 post-immunization. Plasma levels of (**A**) osteoprotegerin (OPG), (**B**) sclerostin (SOST) and (**C**) SOST/OPG ratio. Results are expressed as means ± SEM (n = 8–10 rats/group). ns: non-significant.

To understand the mechanism explaining the vascular effect of ice cryotherapy, absolute number of blood leukocytes was measured. As shown in Fig. 4A–G, ice cryotherapy treatment did not change the circulating leukocyte populations studied. To explore if the vascular effects of ice cryotherapy could be explained by a crosstalk between bones and vessels^22^, blood levels of OPG and SOST were measured. Results showed no difference in OPG and SOST levels (Fig. 5A,B) nor in SOST:OPG ratio between AIA rats treated or not with local cryotherapy (Fig. 5C). Plasma levels of OPG or SOST were not associated with arthritis score or radiographic score in AIA rats (data not shown). No correlation was found between plasma levels of OPG or SOST and circulating leukocytes populations, while a negative correlation was found between plasma levels of OPG and aortic Tc17 cells (r = − 0.5151; *p* = 0.0431 n = 16).

## Discussion

Using the arthritis AIA model in rats, our findings demonstrated the capacity of local ice cryotherapy applied on joints to exert remote effects on a large artery, including a reduction in vascular inflammation and immune cell infiltration, along with a positive effect on arthritis severity and joint damage.

Local cryotherapy is widely used to relieve pain and inflammation in injuries and inflammatory conditions, including RA. It consists in the application on the joints of ice, ice water, cold packs, wet towels or cold gas. The use of cold gas presents challenges in maintaining consistent parameters such as spraying distance and duration, which may affect reproducibility. Conversely, direct local ice application is easier to standardize, induced a deeper temperature drop as compared to cold gas^23^, and did not compromise cartilage integrity^24,25^. In a study investigating the effects of a single cryotherapy session in patients with knee arthritis^8^, no significant differences were observed between ice packs and cold gas in terms of synovial Doppler activity or patient-reported pain, but notable differences emerged regarding inflammatory biomarkers. Ice therapy led to a reduction in synovial pro-inflammatory cytokines, NF-κB-p65, VEGF, and PGE2 levels whereas cold gas only significantly reduced VEGF. In general, ice cryotherapy is better tolerated by patients compared to alternative modalities^26,27^. In the present study, ice sticks placed at the bottom of the cage resulted in an overall reduction of − 65% in rat skin temperature, i.e. a reduction of ~ 10 °C, which is consistent with (or even superior) to the protocols used in humans^23–26,28^. Under these conditions, our data showed that a 14 days- treatment with ice cryotherapy rapidly and robustly reduced arthritis severity and radiographical damage. This result is consistent with a previous animal study^16^ and with some^29^, but not all clinical studies in RA^7^. Studies on the effect of local cryotherapy on disease activity in RA remain limited, inconsistent, and subject to numerous biases, particularly due to concomitant use of anti-inflammatory medications and lack of protocol standardization^4^.

A new finding of this study is the positive effect of ice cryotherapy on endothelial dysfunction in a large vessel such as aorta. The presence of endothelial dysfunction, a condition preceding atherosclerosis development, has been largely documented in patients with RA^30^. Previous studies in the AIA model have identified high vascular levels of COX-2, NAPDH oxidase and arginase-2 as pivotal mechanisms involved in RA-related endothelial dysfunction^31^. Here, local cryotherapy was able to decrease vascular mRNA expression of COX-2 and the NADPH oxidase subunit p47phox, with a non-significant trend for the reduction in arginase-2 expression. It is noteworthy that these effects are similar to those observed in the same animal model with anti-rheumatic drugs such as anti-TNFα^31^ or methotrexate^32^. Somewhat surprising, no reduction of vascular markers of endothelial activation was observed after cryotherapy treatment. The probable reason is that endothelial activation is an event that precedes endothelial dysfunction in the AIA model^13^, like in patients with RA^33^, making our treatment protocol likely too late to change these parameters. VCAM-1 mRNA expression was however increased by ice therapy, as previously observed in AIA rats treated with local cold gas^13^. We hypothesize that such an increase might reflect the initiation of the resolution of inflammation, through the recruitment of resolving macrophage exerting efferocytosis^34^, or of regulatory T cells^35^.

A novel finding is the effect of local ice cryotherapy on aortic wall leukocyte infiltration. In patients with RA, 18F-FDG-PET/CT imaging studies revealed a significant aortic wall infiltration, higher in early compared to established disease^36^. Whereas a study showed that a 6-month-anti-inflammatory treatment reduced aortic wall infiltration^37^, another study reported that patients with RA in clinical remission still have increased inflammation in the arterial wall, despite the use of anti-TNFα therapy^38^. This latter data highlights the interest in finding add-on therapies able to reduce vascular inflammation. Using flow cytometry in aortic tissue, our study demonstrated that ice cryotherapy reduced markedly immune cell infiltration within the aortic wall. This effect was particularly notable for CD4^+^, CD8^+^ T and Tc17 cells, *i*.*e*., lymphocyte subsets that are critically involved in atherosclerosis and vascular structural changes^39^. Mechanistically, the simplistic view that fewer aortic leukocytes infiltration could be the result of fewer circulating leukocytes is wrong, as no difference in circulating leukocyte populations was observed between ice-treated and untreated AIA rats. This suggested that the protective effect conferred by cryotherapy against immune infiltration is mediated by other pathways than a systemic immunosuppressive effect. As ice cryotherapy induced strong effect on joint damage, we hypothesized that a pathway may involve two bone metabolism markers, OPG and SOST. Indeed, data in patients with RA suggested that high levels of OPG and low levels of SOST could be deleterious for vascular health^20,21,40^. Unfortunately, no significant difference in plasma levels of these two markers was observed between AIA and AIA-ice treated rats. Finally, as suggested by studies in RA in which aortic wall infiltration correlated with disease activity (DAS28)^36^, we found that aorta immune infiltration in AIA rats positively correlated with arthritis score, highlighting a potential pivotal role of the anti-inflammatory effects of local ice cryotherapy to explain its remote vascular effects.

Our study presents some limitations. First, the data provided in this present work do not allow for a more precise description of the mechanism by which ice cryotherapy reduces vascular inflammation. Further investigations are necessary to decipher the molecular and cellular mechanisms involved in vascular benefit of ice cryotherapy, as well as its effect on endothelial function or atherosclerotic remodelling process. Second, our study was conducted exclusively in male rats to minimize sex-related variability in clinical and immunoinflammatory responses and thus more clearly characterize the treatment effect. However, because RA predominantly affects women, evaluating the effects of local ice cryotherapy in female animals will be essential to fully conclude on its beneficial effects. Finally, since incorporating a comparative arm in local ice cryotherapy studies is challenging, it would be valuable to compare its anti-inflammatory and vasculoprotective effects with conventional and/or biologic Disease Modifying AntiRheumatic Drugs (e.g., methotrexate, infliximab, tocilizumab) and to investigate whether cryotherapy might potentiate these pharmacological treatments.

In summary, this study highlights a putative remote anti-inflammatory and vasculoprotective effect of local ice cryotherapy in a preclinical model of arthritis. This response involved a reduction of vascular oxidative stress as well as a decrease in immune infiltration cells. The beneficial vascular effects appear to be independent of circulating leukocytes changes. While additional studies are needed to elucidate the mechanisms driving these vascular responses, these findings support ice cryotherapy as a promising and well-tolerated adjunctive intervention for patients with RA.

## Supplementary Information

Supplementary Information.

## Data Availability

The datasets used and/or analyzed during the current study are available from the corresponding author on reasonable request.

## References

[1] Gualtierotti, R., Ughi, N., Marfia, G. & Ingegnoli, F. Practical management of cardiovascular comorbidities in rheumatoid arthritis. *Rheumatol. Ther.* **4**, 293–308 (2017). 28752316 10.1007/s40744-017-0068-0 PMC5696280

[2] Yang, X., Chang, Y. & Wei, W. Endothelial dysfunction and inflammation: Immunity in rheumatoid arthritis. *Mediat. Inflamm.* **2016**, 1–9 (2016). 10.1155/2016/6813016 PMC4829719 27122657

[3] Di Franco, M., Lucchino, B., Conti, F., Valesini, G. & Spinelli, F. R. Asymmetric dimethyl arginine as a biomarker of atherosclerosis in rheumatoid arthritis. *Mediat. Inflamm.* **2018**, 3897295 (2018). 10.1155/2018/3897295 PMC5822828 29576746

[4] Guillot, X. et al. Cryotherapy in inflammatory rheumatic diseases: A systematic review. *Expert Rev. Clin. Immunol.* **10**, 281–294 (2014). 24345205 10.1586/1744666X.2014.870036

[5] Peres, D. et al. The practice of physical activity and cryotherapy in rheumatoid arthritis: Systematic review. *Eur. J. Phys. Rehabil. Med.* **53**, 775–787 (2017). 27996221 10.23736/S1973-9087.16.04534-2

[6] Księżopolska-Orłowska, K. et al. Complex rehabilitation and the clinical condition of working rheumatoid arthritis patients: Does cryotherapy always overtop traditional rehabilitation?. *Disabil. Rehabil.* **38**, 1034–1040 (2016). 26853597 10.3109/09638288.2015.1060265

[7] Hirvonen, H. E., Mikkelsson, M. K., Kautiainen, H., Pohjolainen, T. H. & Leirisalo-Repo, M. Effectiveness of different cryotherapies on pain and disease activity in active rheumatoid arthritis. A randomised single blinded controlled trial. *Clin. Exp. Rheumatol.* **24**, 295–301 (2006). 16870097

[8] Guillot, X. et al. Cryotherapy decreases synovial Doppler activity and pain in knee arthritis: A randomized-controlled trial. *Jt. Bone Spine* **84**, 477–483 (2017). 10.1016/j.jbspin.2016.09.004 27825572

[9] Douzi, W. et al. 1H-NMR-based analysis for exploring knee synovial fluid metabolite changes after local cryotherapy in knee arthritis patients. *Metabolites* **10**, 460 (2020). 33202890 10.3390/metabo10110460 PMC7696760

[10] Guillot, X. et al. Local ice cryotherapy decreases synovial interleukin 6, interleukin 1β, vascular endothelial growth factor, prostaglandin-E2, and nuclear factor kappa B p65 in human knee arthritis: A controlled study. *Arthritis Res. Ther.* **21**, 180 (2019). 31362785 10.1186/s13075-019-1965-0 PMC6668066

[11] Barzegar, M., Babakhani, F., Balochi, R. & Hatefi, M. *Effect of Topical Cooling with Ice and Cold Spray on Knee Joint Position Sense of Athletes with Patellofemoral Pain Syndrome*. https://brieflands.com/articles/jcrps-109762#abstract (2021).

[12] Im, Y.-G. et al. Comparison of changes in facial skin temperature caused by ethyl chloride spraying, ice block rubbing and cold gel packing in healthy subjects. *J. Oral Rehabil.* **39**, 931–940 (2012). 22994138 10.1111/joor.12007

[13] Peyronnel, C. et al. Effects of local cryotherapy on systemic endothelial activation, dysfunction, and vascular inflammation in adjuvant-induced arthritis (AIA) rats. *Arthritis Res. Ther.* **24**, 97 (2022). 35488311 10.1186/s13075-022-02774-1 PMC9052534

[14] Kessler, J. et al. Animal models to study pathogenesis and treatments of cardiac disorders in rheumatoid arthritis: Advances and challenges for clinical translation. *Pharmacol. Res.* **170**, 105494 (2021). 34139344 10.1016/j.phrs.2021.105494

[15] Mossiat, C. et al. Association between arthritis score at the onset of the disease and long-term locomotor outcome in adjuvant-induced arthritis in rats. *Arthritis Res. Ther.* **17**, 184 (2015). 26183428 10.1186/s13075-015-0700-8 PMC4506462

[16] Guillot, X. et al. Local cryotherapy improves adjuvant-induced arthritis through down-regulation of IL-6/IL-17 pathway but independently of TNFα. *PLoS ONE* **12**, e0178668 (2017). 28759646 10.1371/journal.pone.0178668 PMC5536266

[17] Ackerman, N. R. et al. Effects of naproxen on connective tissue changes in the adjuvant arthritic rat. *Arthritis Rheum.* **22**, 1365–1374 (1979). 518718 10.1002/art.1780221208

[18] Totoson, P., Maguin-Gaté, K., Prati, C., Wendling, D. & Demougeot, C. Mechanisms of endothelial dysfunction in rheumatoid arthritis: Lessons from animal studies. *Arthritis Res. Ther.* **16**, R22 (2014). 10.1186/ar4450 PMC3978571 24457026

[19] Peyronnel, C., Totoson, P., Martin, H. & Demougeot, C. Relevance of circulating markers of endothelial activation for cardiovascular risk assessment in rheumatoid arthritis: A narrative review. *Life Sci.*10.1016/j.lfs.2022.121264 (2022). 36470540 10.1016/j.lfs.2022.121264

[20] Nava-Valdivia, C. A. et al. Assessment of serum sRANKL, sRANKL/OPG ratio, and other bone turnover markers with the estimated 10-year risk of major and hip osteoporotic fractures in rheumatoid arthritis: A cross-sectional study. *Biomed. Res. Int.* **2021**, 5567666 (2021). 34497849 10.1155/2021/5567666 PMC8421166

[21] Shui, X. et al. Association of serum sclerostin and osteoprotegerin levels with the presence, severity and prognosis in patients with acute myocardial infarction. *BMC Cardiovasc. Disord.* **22**, 213 (2022). 35546224 10.1186/s12872-022-02654-1 PMC9092859

[22] Vergatti, A. et al. The bone-heart axis in the pathogenesis of cardiovascular diseases: A narrative review. *Nutr. Metab. Cardiovasc. Dis.* **35**, 103872 (2025). 39956695 10.1016/j.numecd.2025.103872

[23] de Estéfani, D. et al. Volume of water added to crushed ice affects the efficacy of cryotherapy: A randomised, single-blind, crossover trial. *Physiotherapy* **107**, 81–87 (2020). 32026839 10.1016/j.physio.2019.12.005

[24] Ho, S. S. et al. Comparison of various icing times in decreasing bone metabolism and blood flow in the knee. *Am. J. Sports Med.* **23**, 74–76 (1995). 7726354 10.1177/036354659502300112

[25] Kanlayanaphotporn, R. & Janwantanakul, P. Comparison of skin surface temperature during the application of various cryotherapy modalities. *Arch. Phys. Med. Rehabil.* **86**, 1411–1415 (2005). 16003673 10.1016/j.apmr.2004.11.034

[26] Fachin, K. D. S. M. et al. Comparison of cryotherapy performed with ice or gel and superficial skin cooling of older women: A randomized, crossover. *Clin. Trial. J Geriatr. Phys. Ther.*10.1519/JPT.0000000000000412 (2024). 10.1519/JPT.0000000000000412 38502943

[27] Straub, R. H. et al. Acute cold stress in rheumatoid arthritis inadequately activates stress responses and induces an increase of interleukin 6. *Ann. Rheum. Dis.* **68**, 572–578 (2009). 18413439 10.1136/ard.2008.089458

[28] Leite, M. & Ribeiro, F. Liquid ice fails to cool the skin surface as effectively as crushed ice in a wet towel. *Physiother. Theory Pract.* **26**, 393–398 (2010). 20658925 10.3109/09593980903229240

[29] Jastrząbek, R., Straburzyńska-Lupa, A., Rutkowski, R. & Romanowski, W. Effects of different local cryotherapies on systemic levels of TNF-α, IL-6, and clinical parameters in active rheumatoid arthritis. *Rheumatol. Int.* **33**, 2053–2060 (2013). 23397259 10.1007/s00296-013-2692-5

[30] Bordy, R. et al. Microvascular endothelial dysfunction in rheumatoid arthritis. *Nat. Rev. Rheumatol.* **14**, 404–420 (2018). 29855620 10.1038/s41584-018-0022-8

[31] Totoson, P., Maguin-Gaté, K., Nappey, M., Wendling, D. & Demougeot, C. Endothelial dysfunction in rheumatoid arthritis: Mechanistic insights and correlation with circulating markers of systemic inflammation. *PLoS ONE* **11**, e0146744 (2016). 26761790 10.1371/journal.pone.0146744 PMC4711944

[32] Bordy, R. et al. Methotrexate did not improve endothelial function in rheumatoid arthritis: a study in rats with adjuvant-induced arthritis. *Clin. Exp. Rheumatol.* **37**, 81–88 (2019). 30148435

[33] Södergren, A. et al. Atherosclerosis in early rheumatoid arthritis: Very early endothelial activation and rapid progression of intima media thickness. *Arthritis Res. Ther.* **12**, R158 (2010). 20712865 10.1186/ar3116 PMC2945061

[34] Gerlach, B. D. et al. Efferocytosis induces macrophage proliferation to help resolve tissue injury. *Cell Metab.* **33**, 2445-2463.e8 (2021). 34784501 10.1016/j.cmet.2021.10.015 PMC8665147

[35] Piao, W. et al. PD-L1 signaling selectively regulates T cell lymphatic transendothelial migration. *Nat. Commun.* **13**, 2176 (2022). 35449134 10.1038/s41467-022-29930-0 PMC9023578

[36] Agca, R. et al. Arterial wall inflammation is increased in rheumatoid arthritis compared with osteoarthritis, as a marker of early atherosclerosis. *Rheumatology (Oxford)*10.1093/rheumatology/keaa789 (2021). 33447846 10.1093/rheumatology/keaa789 PMC8516502

[37] Blanken, A. B. et al. Arterial wall inflammation in rheumatoid arthritis is reduced by anti-inflammatory treatment. *Semin. Arthritis Rheum.* **51**, 457–463 (2021). 33770536 10.1016/j.semarthrit.2021.03.008

[38] Bernelot Moens, S. J. et al. Unexpected arterial wall and cellular inflammation in patients with rheumatoid arthritis in remission using biological therapy: A cross-sectional study. *Arthritis Res. Ther.* **18**, 115 (2016). 27209093 10.1186/s13075-016-1008-z PMC4875657

[39] Saigusa, R., Winkels, H. & Ley, K. T cell subsets and functions in atherosclerosis. *Nat. Rev. Cardiol.* **17**, 387–401 (2020). 32203286 10.1038/s41569-020-0352-5 PMC7872210

[40] Genre, F. et al. Implication of osteoprotegerin and sclerostin in axial spondyloarthritis cardiovascular disease: Study of 163 Spanish patients. *Clin. Exp. Rheumatol.* **36**, 302–309 (2018). 29303699

