# Supplementary material for: Local ice cryotherapy reduced vascular inflammation in large artery from rats with arthritis
Source: Sci Rep. 2026 Mar 30;16:10599. doi: 10.1038/s41598-026-41594-0 (PMC13039691; doi:10.1038/s41598-026-41594-0)
Supplement: Supplementary file 1 — Supplementary Information. [file 41598_2026_41594_MOESM1_ESM.docx]

**Local ice cryotherapy reduced vascular inflammation in large artery from rats with arthritis**

Célian Peyronnel^a^, Perle Totoson^a,#^, Maude Tournier^a^, Francis Bonnefoy^a^, Xavier Guillot^b^, Philippe Saas^c^, Frank Verhoeven^a^, Hélène Martin^a^, Céline Demougeot^a^

^a^ Université Marie et Louis Pasteur, EFS, INSERM, UMR 1098 RIGHT, F-25000 Besançon, France

^b^ Service de Rhumatologie, CHU Felix Guyon, Saint-Denis, Ile de la Réunion, France

^c^ EFS AuRA, Univ. Grenoble-Alpes, INSERM U1209, CNRS UMR5309, Institute for Advanced Biosciences, F-38000 Grenoble, France

## ^#^ Corresponding author:

Dr. Perle Totoson

19 rue Ambroise Paré, bâtiment S, 25030 BESANCON cedex, FRANCE.

Tel: (33) 3 63 08 23 40

E-mail address: perle.totoson@univ-fcomte.fr

**Running title:** *Local ice cryotherapy reduced aortic inflammation in arthritis*

**Supplemental Figure**

**
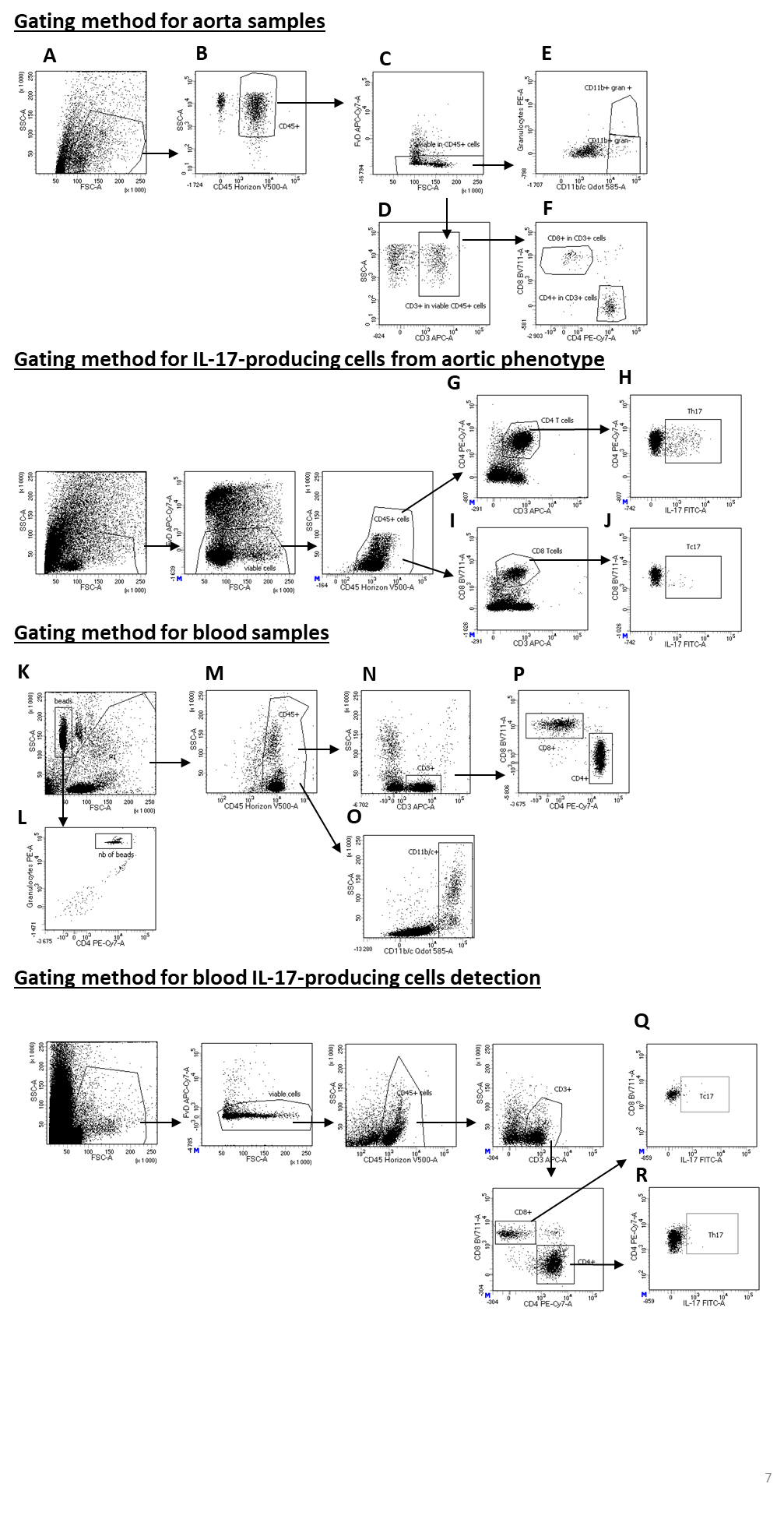
**

**Supplemental Figure 1. Gating strategy example used to sort leukocyte subpopulations in blood and thoracic aorta samples in AIA rats by flow cytometry.**

Aortic and blood leukocytes subpopulations of AIA rats were sorted by flow cytometry according to the strategy described below. Different leukocyte subpopulations were determined using membrane labeling (CD45^+^ leukocytes, CD11b/c^+^ monocytes/macrophages, T lymphocytes CD4^+^ and CD8^+^, granulocytes (RP-1^+^) or intracellular (anti-IL-17A antibody). **In aortic samples**: leukocytes were isolated according to their size, granularity **(A)** and CD45^+^ labeling **(B)** and only the viable cells **(C)** (labeled with fixable dead cell stain) were isolated. Viable leukocytes (CD45^+^) were separated into T lymphocytes (CD3^+^) **(D)** and innate immune cells (CD11b/c^+^) **(E)** according to their labeling. Among CD11b/c^+^, neutrophils (RP-1^+^) and monocytes/macrophages (RP-1-) could also be dissociated **(E)**. Among the T lymphocytes (CD3^+^), the CD4^+^ and CD8^+^ T cells were separated according to their respective labeling **(F)**. Intracellular labeling of IL-17A allowed to separate CD4^+^ and CD8^+^ T cells producing IL-17A (Th17 and Tc17) or not **(G-J)**. **In blood samples:** the same methodology was followed with an additional preliminary step. During the sorting of leukocytes (CD45^+^), the beads of the Trucount tubes were isolated and counted in order to subsequently find the exact number of labeled cells **(K, L)**. The separation of leukocytes **(M)**, monocytes/macrophages, neutrophils **(O)**, lymphocytes CD4^+^ and CD8^+^ **(N, P)**, Th17 **(R)** and Tc17 **(Q)** in the blood was done in the same way as in the aorta samples.
